# An alternative way of SARS‐COV‐2 to induce cell stress and elevated DNA damage risk in cardiomyocytes without direct infection

**DOI:** 10.1002/iid3.638

**Published:** 2022-06-10

**Authors:** Houqing Zhou, Xiaohu Ren, Yang Yang, Benhong Xu, Yichong Li, Yin Feng, Fang Shisong, Jianjun Liu

**Affiliations:** ^1^ Fuwai Hospital Chinese Academy of Medical Sciences Shenzhen, Nanshan District, Shenzhen Guangdong China; ^2^ Shenzhen Key Laboratory of Modern Toxicology, Shenzhen Medical Key Discipline of Health Toxicology (2020‐2024), Shenzhen Center for Disease Control and Prevention Nanshan District, Shenzhen Guangdong China; ^3^ Pingshan Translationtal Medicine Center Shenzhen Bay Laboratory Center Pingshan District, Shenzhen Guangdong China; ^4^ The Third People's Hospital of Shenzhen Longgang District, Shenzhen Guangdong China

**Keywords:** animals, blood, infections, tissues, viral/retroviral, Human

## Abstract

**Background**: The outbreak of severe acute respiratory syndrome coronavirus 2 (SARS‐COV‐2) in 2020 has led to millions of deaths worldwide. Case reports suggested that infection of SARS‐CoV‐2 is potentially associated with occurrences of cardiovascular pathology. However, the mode of action and mechanisms of SARS‐CoV‐2 influencing cardiomyocytes still remain largely unclear.**Aims**: To explore the mechanisms underlying cardiomyocytes damage induced by SARS‐CoV‐2 infection.**Materials & Methods**: the serum markers of cardiovascular injury were analyzed by ELISA. The isolated SARS‐CoV‐2 virus were co‐cultured with human cardiomyocytes (AC16) and immunofluorescence assay was used evaluate the invasion of virus. Moreover, serum obtained from acute stage of SARS‐CoV‐2 infected patients and healthy controls were used to incubate with AC16 cells, then indicators associated with cell stress and DNA damage were analyzed by Western‐blot.**Results**: we found that high‐sensitivity troponin T (hsTnT), an indicator of cardiovascular disease, was higher in the acute stage of COVID‐19. Additionally, in vitro coculture of SARS‐CoV‐2 and AC16 cells showed almost no infectious ability of SARS‐CoV‐2 to directly infect AC16 cells. Results of serum treatment suggested that serum from infected subjects induced cell stress (upregulation of p53 and HSP70) and elevation of DNA damage risk (increased γH2Ax and H3K79me2) in AC16.**Discussion**: our observations indicated a hard way for SARS‐CoV‐2 to infect cardiomyocytes directly. However, infection‐induced immune storm in serum could bring stress and elevated DNA damage risks to cardiovascular system.**Conclusion**: These findings indicated the possibilities of SARS‐CoV‐2 inducing stress and elevating DNA damage risk to cardiomyocytes without direct infection.

## Graphical abstract

SARS‐COV‐2‐induced cardiovascular pathology could be through indirectly damaging cardiomyocytes.

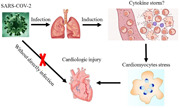

## 1 INTRODUCTION

Severe acute respiratory syndrome coronavirus 2 (SARS‐COV‐2) outbreak was first reported in December 2019, Wuhan, China. The virus caused the highly infectious disease referred to as SARS‐COV‐2. Infection of SARS‐COV‐2 has now spread worldwide and has become a global pandemic affecting millions of people.^1^ SARS‐CoV‐2 is highly transmissible with a broad tissue injury including an incidence of arrhythmia.^2^ SARS‐CoV‐2 preferentially infects the low respiratory tract but is also a virus with multiorgan tropism.^3^ There are data pointing out an elevation of cardiovascular disease morbidity and mortality in subjects after infection of SARS‐CoV‐2 (approximately 12% of SARS‐CoV‐2 patients undergoing acute cardiac injury).^4^ However, important questions remain unclear regarding the effect of SARS‐CoV‐2 on the cardiac system. A cohort study analyzed 39 consecutive autopsy cases, between which 16 were found SARS‐CoV‐2‐positive within the myocardium.^5^ A cardiovascular magnetic resonance imaging analysis was conducted on recently recovered subjects undergone SARS‐CoV‐2 infection and ongoing myocardial inflammation with detectable (above 3 pg/ml) high‐sensitivity troponin T (hsTnT), the findings indicated the need for investigation of the long‐term cardiovascular consequences after SARS‐CoV‐2.^6^ There was another retrospective study that suggested an increased risk of cardiovascular diseases, such as chronic heart failure, congenital heart disease, and chronic obstructive pulmonary disease in children infected with SARS‐CoV‐2.^7^ Although many observations suggested the potential of SARS‐CoV‐2 inducing cardiomyocytes injury, the mode of action and mechanisms still remain largely unknown. Some theory indicates that a high inflammatory burden could be a consequence of SARS‐CoV‐2 infection, which potentially induces vascular inflammation, myocarditis, and cardiac arrhythmias.^8^ One of the consequences of inflammatory burdens in SARS‐CoV‐2‐induced acute respiratory disease is the so‐called “cytokine storm”; excessive pro‐inflammatory cytokines were produced and released into the circulation of blood leading to multiorgan failure and even death.^9^, ^10^ Furthermore, the inflammatory cytokines could induce DNA damage and even inhibition of associated DNA repair.^11^ Another research reported that both cardiomyocytes, vascular endothelium, cardiac fibroblasts, pericytes, and vascular smooth cells express angiotensin‐converting enzyme 2 (ACE2) at respective levels.^12^, ^13^ However, whether SARS‐CoV‐2 directly proliferates in the cardiomyocytes is still under debate. In this study, we collected serum from 18 SARS‐CoV‐2‐infected subjects and evaluated levels of high‐sensitivity cardiac troponin T (hs‐cTnT) and N‐terminal brain natriuretic peptide (NT‐proBNP). Moreover, isolated virus and in vitro‐cultured human cardiomyocyte cells (AC16) were cocultured to evaluate the susceptibility of cardiomyocytes to SARS‐CoV‐2 infection. Serum (from SARS‐CoV‐2 diagnosed subjects) treatment to AC16 cells was used to analyze the possible mode of action for SARS‐CoV‐2‐induced cardiovascular stress.

## 2 MATERIALS AND METHODS

### 2.1 Cells and reagents

The human cardiomyocyte cells (AC16) were obtained from Merk (Belize). The human lung cells (BEAS‐2B), bronchial cells (,16‐HBE) and lung cancer cells (A549) were from previously cryopreservation in our lab. Vero cells and Pierce BCA protein quantification kit were obtained from Thermo Fisher Scientific. High‐sensitivity cardiac troponin T (hs‐cTnT) and N‐terminal brain natriuretic peptide (NT‐proBNP) ELISA kits were purchased from R&D. Primary antibodies were obtained from Abcam and secondary antibodies were obtained from Santa Cruz. Tris‐buffered saline containing 0.05% Tween‐20 (TBST) was purchased from Double‐helix. Bovine serum albumin (BSA) was purchased from Sigma‐Aldrich. Alexa fluor 488‐labeled goat anti‐human IgG antibody was obtained from Jackson. Tris‐buffered saline containing 0.05% Tween‐20 (TBST) was purchased from double‐helix. Tetramethylbenzidine dihydrochloride (TMB) and sulfuric acid were purchased from Sangon. RNeasy Mini Kit was obtained from Qiagen, and SYBRGreen master mixture and PrimeScript™ RT Reverse Transcript Kit were both obtained from Takara. The primers used in this study were synthesized by Sangon.

### 2.2 Experimental ethics policy

This study was approved by the Ethics Committees of Shenzhen Center for Disease Control and Prevention (approval number: 2020 025A). All the experiments were performed in BSL‐3 facilities before the virus was inactivated in accordance with the management practices.

### 2.3 Clinical samples

Serum samples from 18 subjects with SARS‐CoV‐2 infection were collected from The Third People's Hospital of Shenzhen. Three subjects were asymptomatic, 10 subjects were moderate, and 5 subjects were severe. Moreover, serum from each subject was collected in an acute stage (1 or 2 days after admission) and in a recovery stage (1 or 2 days before discharge), respectively. Nineteen healthy controls were selected from our previously established cohorts.^14^ All the experiments were performed in BSL‐3 facilities before the virus was inactivated in accordance with the management practices. The collection and handling of serums from SARS‐COV‐2 infections were approved by the Ethics Committee of Shenzhen Center for Disease Control and Prevention.

### 2.4 Enzyme‐linked immunosorbent assay assay

Enzyme‐linked immunosorbent assay (ELISA) analysis for hs‐cTnT and NT‐proBNP were performed in the BSL‐3 laboratory and followed the manufacturer's instructions.

### 2.5 Virus isolation and host cell infection assay

The virus isolation was conducted as previously reported.^15^ Briefly, the bronchoalveolar lavage fluid was collected from the patient 1 day after admission. The suspensions containing the SARS‐COV‐2 virus were separated by centrifugation at 5000 rpm, 4°C for 5 min. Two hundred milliliters of supernatant were incubated with Vero cells in a six‐well plate at 37°C and 5% CO_2_ for 1 h in a biosafety level 3 (BSL‐3) laboratory. The cells were then washed with PBS 3 and continuously cultured with fresh DMEM containing 2% fetal bovine serum (FBS) and 1% penicillin–streptomycin. The supernatant was harvested when viral RNA was detected at 6 dpi by RT‐PCR. The AC16 cells were cultured in DMEM/F12 medium supplemented with 12% FBS, 100 μg/ml streptomycin, and 100 units/ml penicillin at 37°C with 5% CO_2_. In all, 0.05 MOI (multiplicity of infection) isolated viruses were cocultured with either AC16 cells or Vero cells (positive control) for 24 h in a six‐well culture plate with a cover slide in the bottom.

### 2.6 Indirect cell immunofluorescence assay

After infection, AC16 and Vero cells were fixed in 4% formaldehyde for 1 h at room temperature after infection. Cells were permeabilized in 0.5% Triton X‐100 and blocked in 5% BSA in PBS. The cells were then probed with the serum of the patient or healthy control at a dilution of 1:1000 for 1 h at room temperature. After three times of wash with PBS, cells were incubated with goat anti‐human IgG antibody conjugated with Alexa fluor 488 at a dilution of 1:1000 for 1 h (Invitrogen). The cells were then washed and stained with Hoechest‐33342 (Invitrogen) to detect nuclei. Fluorescence images were acquired by a TCS SP5 confocal microscopy (Leica Microsystems).

### 2.7 Treatment of serum obtained from subjects in an acute stage

The AC16 cells were cultured in DMEM/F12 medium, 100 μg/ml streptomycin, and 100 units/ml penicillin at 37°C with 5% CO_2_ for 24 h in a 12‐well plate. The negative control cells were supplemented with 10% fetal bovine serum and serum from healthy controls, respectively. The treated cells were supplemented with 10% serum obtained from subjects in an acute stage (serum from five subjects was pooled).

### 2.8 Transcriptional levels of ACE2 and TMPRSS2 by RT‐PCR

The primers against two major receptors of the SARS‐CoV‐2 virus, ACE2 (F: CTTTCTGCAGCCACACCTAAGC, R: GCAGAGTCCCAACAATCGTGAG) and Transmembrane protease serine 2 (TMPRSS2) (F: CCTGTGTGCCAAGACGACTG, R: CTGGTGGATCCGCTGTCATC) were designed by DNAsist 3.1.0 and evaluated by BLAST. The total RNAs of BEAS‐2B, A549, 16HBE, and AC16 cells were extracted by an RNeasy kit, reverse‐transcribed by a One Step PrimeScript™ RT‐PCR kit (Takara). Partial CDS of ACE2 and TMPRSS2 were amplified and then analyzed by an Applied Biosystems™ 7500 Real‐Time PCR system.

### 2.9 Western blot analysis

The serum‐treated AC16 cells were harvested and total protein was extracted. Protein concentration in each group was determined using the Micro BCA Protein Assay Kit (Thermo Fisher Scientific). Twenty‐five micrograms of protein per group were separated on 10% SDS‐PAGE gels and transferred to PDVF membranes. The membranes were blocked by freshly prepared Tris‐buffered saline with 0.1% Tween 20 (TBST) containing 5% BSA for 1 h at room temperature. Primary antibodies against phosphorylation at serine 139 of histone H2Ax (γH2Ax), dimethylation at lysine 79 of Histone H3 (H3K79me2), Tumor protein 53 (p53), and HSP70 (heat‐shock protein 70) were diluted at 1:1000 in TBST, while GAPDH (glyceraldehyde‐3‐phosphate dehydrogenase) were diluted at 1:3000 in TBST. The diluted antibodies were incubated with PDVF membranes overnight at 4°C. After three times washing by TBST, secondary antibodies coupled with horseradish peroxidase were incubated with PDVF membranes for 1 h at room temperature. Images of chemical illuminance were acquired by an ImageQuant 300 RT‐ECL system (GE Healthcare); the gray values of protein bands were obtained and analyzed by the Quantity One software with version 4.6.2 (Bio‐Rad).

### 2.10 Statistical analysis

Two‐sample *t*‐tests were used to determine the significant difference between each group, with a *p *value less than .05 considered significant.

## 3 RESULTS

### 3.1 Clinical characteristics

The severity grade of SARS‐CoV‐2 infection was determined according to the Seventh Edition of China National Health Commission Guidelines for Diagnosis and Treatment of SARS‐CoV‐2 infection. SARS‐CoV‐2 infected subjects (*n* = 18) in this study were hospitalized, including 3 asymptomatic, 10 moderate, and 5 severe subjects. The clinical characteristics are listed in Table 1. Briefly, the median age increased along with severity: 10.3 ± 5.5 (asymptomatic subjects), 36.2 ± 13.2 (moderate subjects), and 61.6 ± 11.0 (severe subjects). Nonetheless, the average systolic/diastolic blood pressure elevated along with along with severity: 113.0 ± 19.0/65.0 ± 21.0 (asymptomatic subjects), 82.1 ± 7.0/82.1 ± 7.0 (moderate subjects), and 87.2 ± 12.0/87.2 ± 12.0 (severe subjects). The heart rate was also elevated along with severity: 81.3 ± 7.4 (asymptomatic subjects), 87.7 ± 9.0 (moderate subjects), and 92.6 ± 5.9 (severe subjects). One‐way ANOVA analysis of clinical characteristic parameters indicated a higher risk of severity along with increasing age (*p* = .002) and systolic (*p* = .04), as listed in Supporting Information: Table 1S

**Table 1:** Clinical characteristics of subjects included in this study.

Severity grade	Age	Sex (males/females)	Blood pressure (systolic/diastolic)	Heart rate (beats per min)	Pulmonary lesions
Asymptomatic	10.3 ± 5.5	1/2	113.0 ± 19.0/65.0 ± 21.0	81.3 ± 7.4	No obvious abnormality in the lungs
Moderate	36.2 ± 13.2	4/5	82.1 ± 7.0/82.1 ± 7.0	87.7 ± 9.0	Multiple bilateral infectious lung nodules; diffuse ground‐glass shadow in two lungs; small pulmonary nodules
Severe	61.6 ± 11.0	2/3	87.2 ± 12.0/87.2 ± 12.0	92.6 ± 5.9	multiple bilateral infectious lung nodules; diffuse ground‐glass shadow in two lungs

### 3.2 ELISA assay revealed that serum levels of hs‐cTnT were higher in an acute stage of SARS‐COV‐2 infection

**Figure 1:**
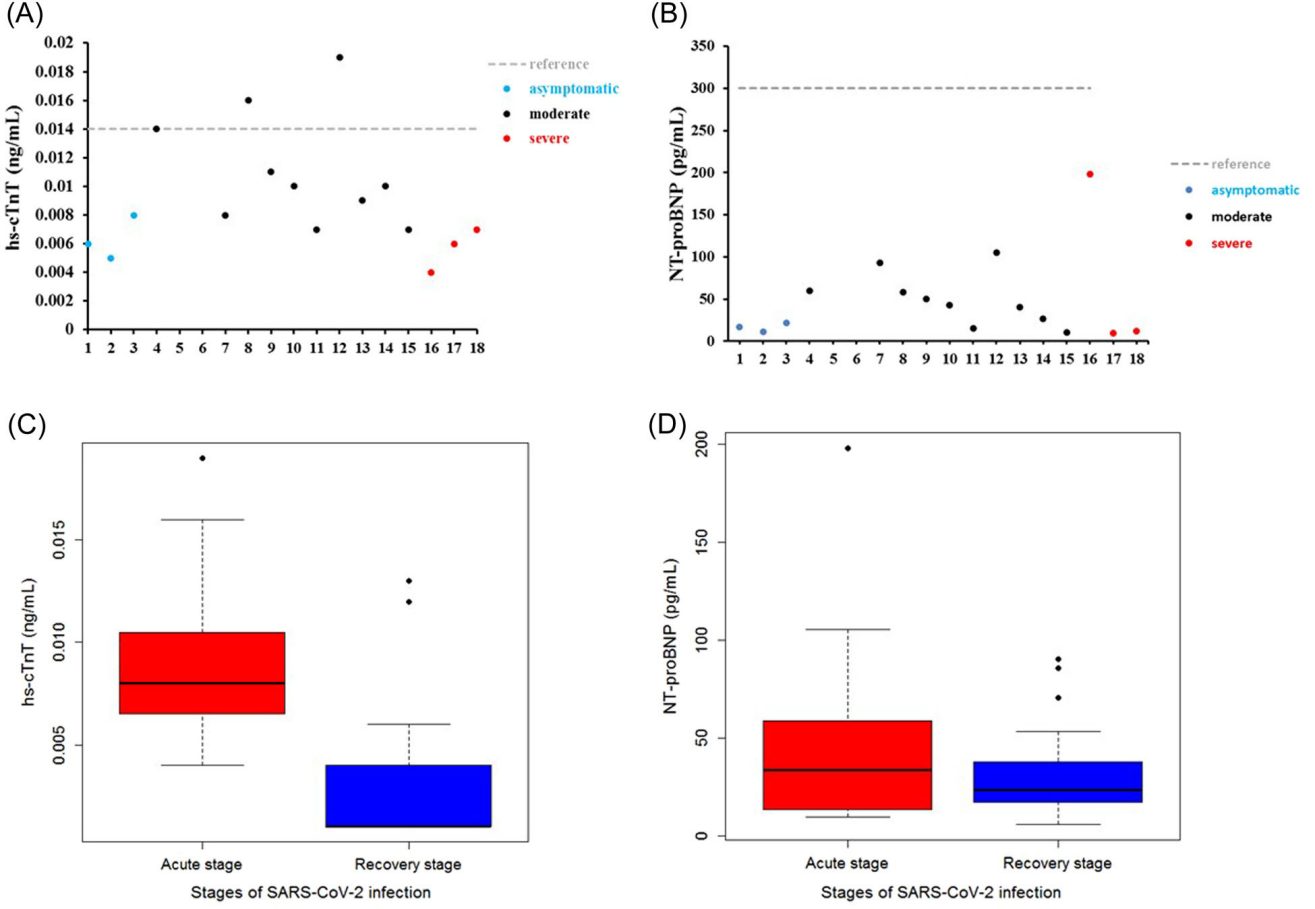
Evaluation of hs‐cTnT and NT‐proBNP in SARS‐CoV‐2 infected subjects. The serum levels of hs‐cTnT and NT‐proBNP were measured by ELISA. (A) Distribution of hs‐cTnT in SARS‐CoV‐2‐infected subjects, red dashed lines indicate reference range, and different colors of dots represent different severe grades (asymptomatic *n* = 3, moderate *n* = 10, severe *n* = 5). (B) Distribution of NT‐proBNP in SARS‐CoV‐2‐infected subjects, red dashed lines represent the reference range, and different colors of dots indicate different severe grades (asymptomatic *n* = 3, moderate *n* = 10, severe *n* = 5). (C) Box‐plot of hs‐cTnT in acute and recovery stages of SARS‐CoV‐2 infection, the black line represents the median, the level of hs‐cTnT was significantly lower in the recovery stage compared with the acute stage, *p* < .05. (D) Box‐plot of NT‐proBNP in acute and recovery stages of SARS‐CoV‐2 infection, the black line represents the median, the level of NT‐proBNP was significantly lower in the recovery stage compared with the acute stage, *p* < .05.

The serum levels of hs‐cTnT and NT‐proBNP were measured in 18 subjects (3 were asymptomatic, 10 were moderate, and 5 were severe) by ELISA assay. The results showed that hs‐cTnT and NT‐proBNP were slightly beyond reference ranges in very few samples (Figure 1A,B). However, further analysis by comparing the acute and convalescent stages suggested that hs‐cTnT was at a relatively higher level in the acute stage of SARS‐COV‐2 infection (Figure 1CD). Additionally, the levels of hs‐cTnT and NT‐proBNP in serum from healthy controls were also measured, results suggested no significant differences between healthy controls and convalescent stages could be observed (Supporting Information: Figure S1). These observations suggested that SARS‐COV‐2 infection could induce increased stress on the cardiovascular system without significant injury.

### 3.3 Inability to infect human cardiomyocytes of SARS‐COV‐2 virus

**Figure 2:**
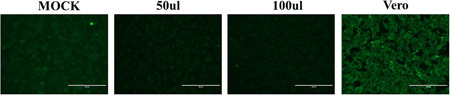
Infectious ability measurement of SARS‐COV‐2 by an indirect cell immunofluorescence assay. The infectious ability of SARS‐COV‐2 to AC16 cells was measured by an indirect cell immunofluorescence assay. MOCK is a negative control without treatment of isolated SARS‐COV‐2; Vero cells were used as a positive control.

**Figure 3:**
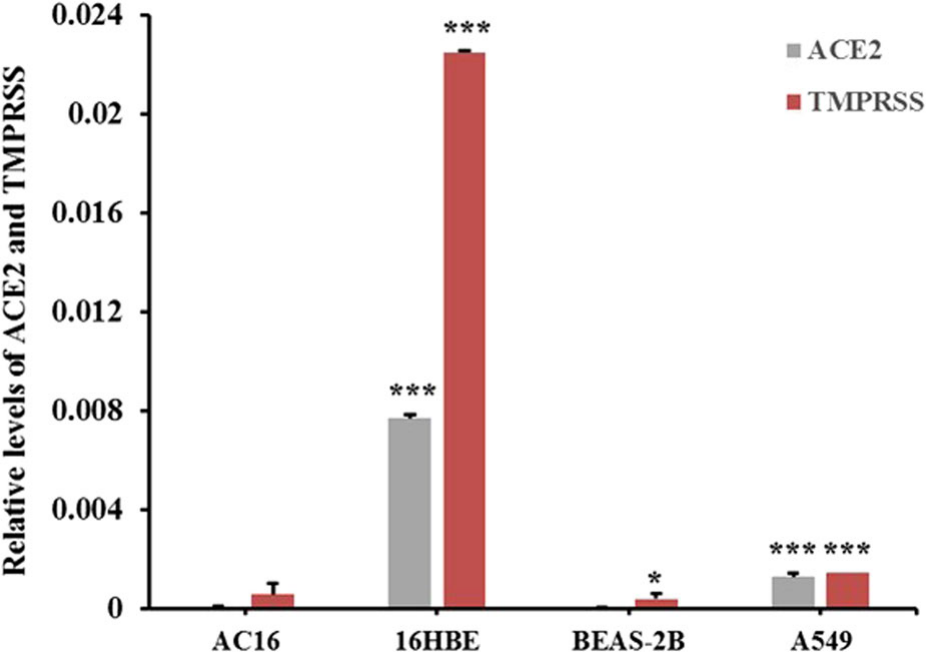
Transcriptional levels of ACE2 and TMPRSS in different cell lines. The transcriptional levels of ACE2 and TMPRSS in AC16, BEAS‐2B, 16HBE, and A549 cells were evaluated by quantitative RT‐PCR analysis. *: compared with AC16 cells, *p* < .05; ***: compared with AC16 cells, *p* < .001.

To further study the way of SARS‐COV‐2 induces cardiac stress, the human cardiomyocyte cells (AC16) were used for the host cell infection analysis. The AC16 cells were incubated with isolated SARS‐COV‐2, and then the positive serum was used to probe the virus inside the cells. Strong fluorescent signals could be observed in Vero cells while hardly can be seen in AC16 cells after incubation with the SARS‐COV‐2 virus (Figure 2). The observation indicated that SARS‐COV‐2 merely directly infects cardiomyocytes. To further evaluate the possible cause of the inability of SARS‐COV‐2 to penetrate the cell membrane of AC16, we analyzed levels of two known receptors (ACE2 and TMPRSS, which were required by SARS‐COV‐2 to invade cells) in different cell types. The results suggested that compared with lung or bronchial cells, ACE2 and TMPRSS were both at a relatively low level in AC16 cells (Figure 3).

### 3.4 Serum from subjects in an acute stage induces upregulation of DNA damage and cell stress markers in AC16 cells

**Figure 4:**
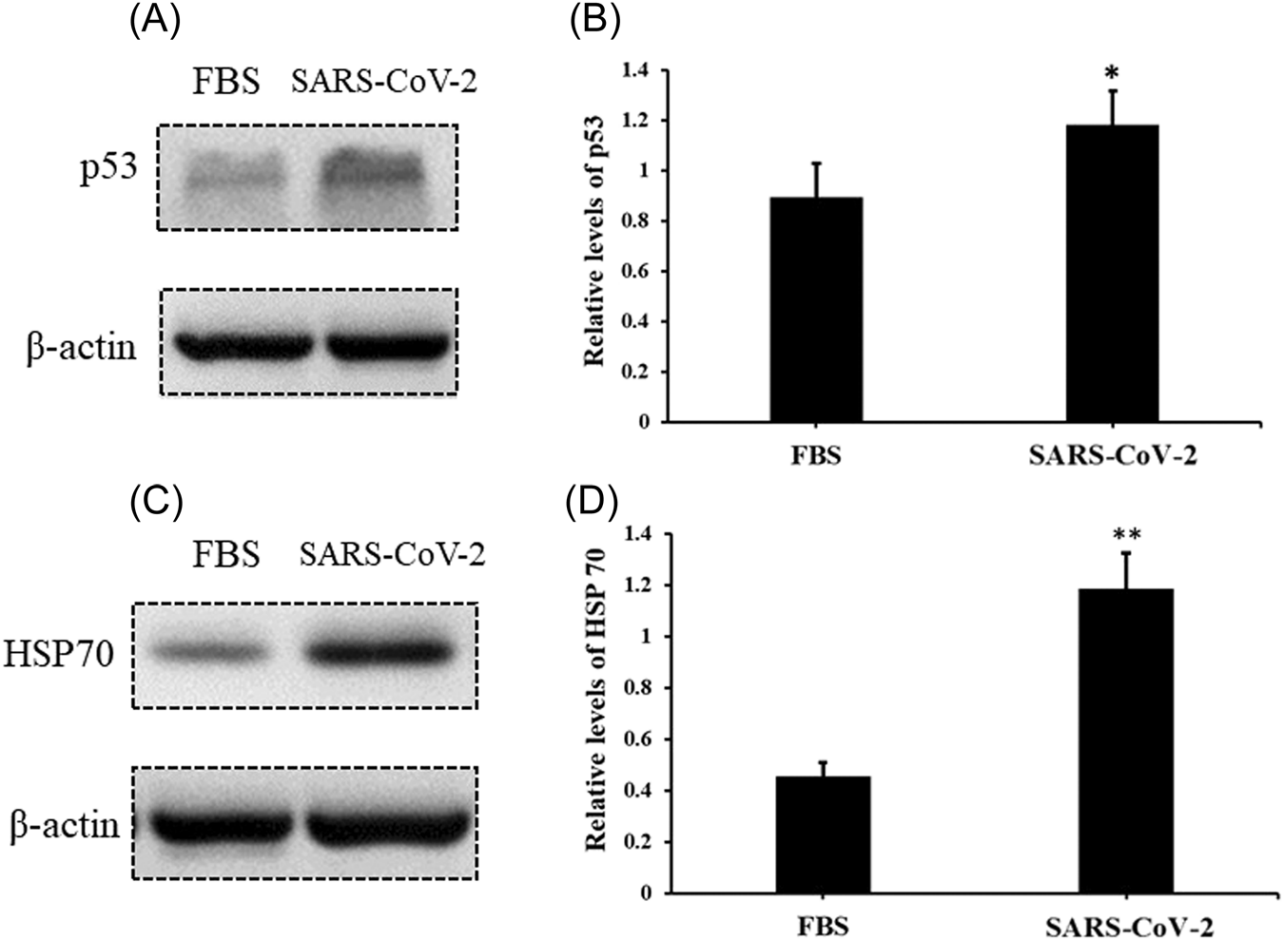
Serum from SARS‐CoV‐2‐infected subjects induces upregulation of p53 and HSP70 in AC16 cells. The relative level of p53 and HSP70 in AC16 cells were evaluated by western blot analysis after treatment of serum from SARS‐CoV‐2‐infected subjects for 24 h. (A) Protein band of p53; (B) the relative level of p53, bar graphics reflect the mean ± SD of at least three independent experiments, *p < .05 compared with control AC16 cells; (C) protein band of HSP70; (D) the relative level of HSP70, bar graphics reflect the mean ± SD of at least three independent experiments, *p < .05 compared with control AC16 cells.

**Figure 5:**
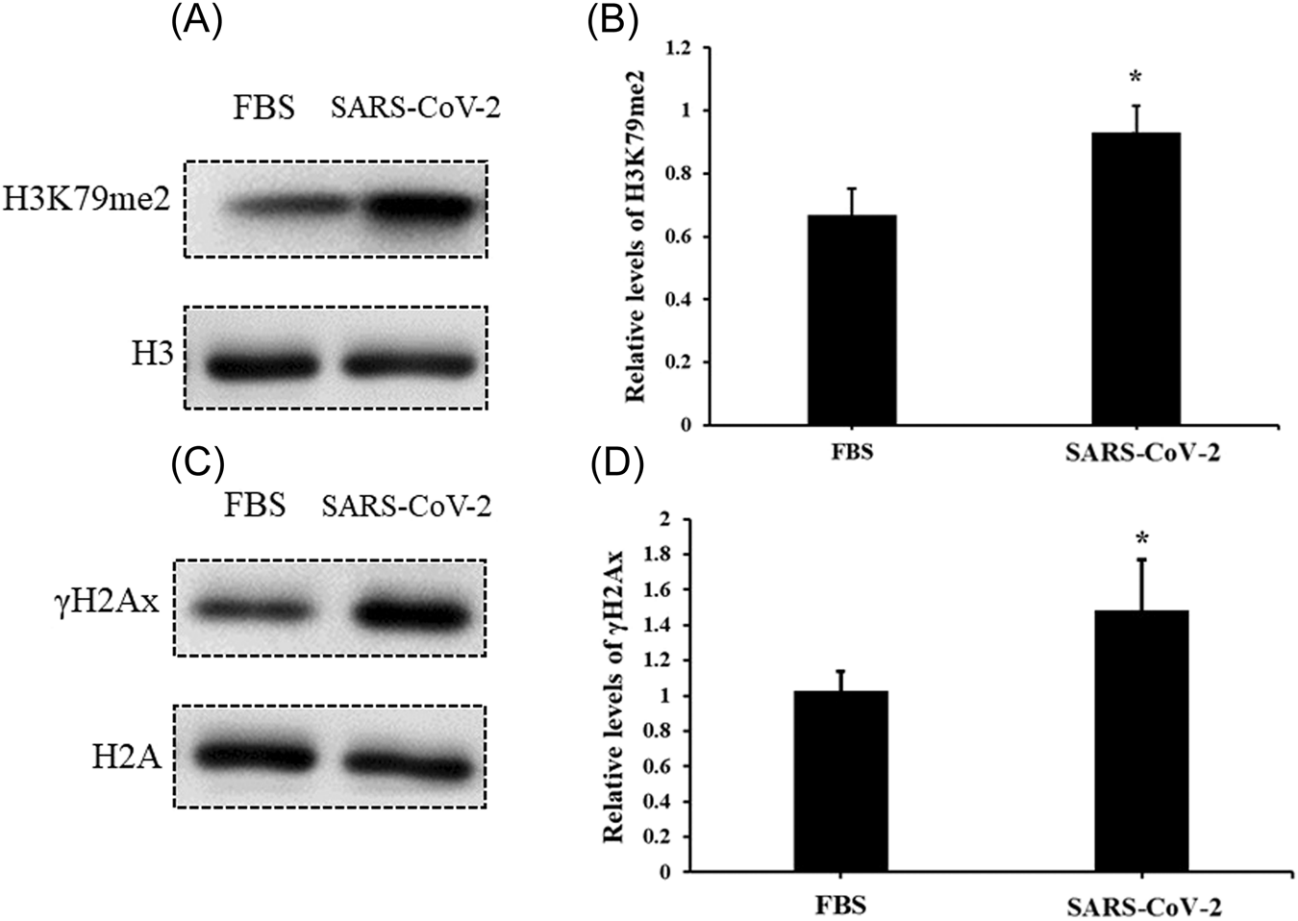
Serum from SARS‐CoV‐2‐infected subjects induces elevation of γH2Ax and H3K79me2 in AC16 cells. The relative level of γH2Ax and H3K79me2 in AC16 cells was evaluated by western blot analysis after treatment of serum from SARS‐CoV‐2‐infected subjects for 24 h. (A) Protein band of γH2Ax; (B) the relative level of γH2Ax, bar graphics reflect the mean ± SD of at least three independent experiments, *p < .05 compared with control AC16 cells; (C) protein band of H3K79me2; (D) the relative level of H3K79me2, bar graphics reflect the mean ± SD of at least three independent experiments, *p < .05 compared with control AC16 cells.

After infection, AC16 cells were collected, and the cell stress markers, such as p53 and HSP70, were evaluated. The results suggested that the levels of stress signal proteins, such as p53 and HSP70, were significantly elevated due to the treatment of serum with SARS‐COV‐2 infection compared to negative controls (Figure 4). Nonetheless, DNA damage is another critical biological event; we further analyzed the markers of DNA damage response. Relative levels of γH2Ax and H3K79me2 were also found upregulated in AC16 cells (Figure 5), which indicates that the incidence of DNA damage events increased under the treatment of serum from SARS‐COV‐2 infection. Nonetheless, to exclude the impacts of human serum, we treated AC16 cells with serum from healthy controls and FBS, and then analyzed levels of p53, HSP70, γH2Ax, and H3K79me2 respectively. However, results showed no significant differences between these treatments (Supporting Information: Figures S2, and S3).

## 4 DISCUSSION

There is still a controversy about the cause of cardiovascular stress or even injury in SARS‐COV‐2 infection. In this study, we analyzed serum levels of hs‐cTnT and NT‐proBNP in 18 enrolled patients. Serum Levels of NT‐proBNP of all subjects were within the reference range and levels of hs‐cTnT were abnormal in only a few subjects. Furthermore, hs‐cTnT was found at a relatively higher level in serum in the acute stage compared with serum in the recovery stage. Although the sample size was limited in this study, these observations still suggested that SARS‐COV‐2 infection potentially induces stress in the cardiovascular system. Thus, we could collect more biological samples to expand the sample size, and further confirm SARS‐COV‐2 infection‐induced cardiovascular burden. To further evaluate the way SARS‐COV‐2 induces cardiovascular stress, we incubated SARS‐COV‐2 with AC16 cells and confirmed the fluorescent signal representing infection by cell immunofluorescence assay. The results indicated that SARS‐COV‐2 may not directly infect human cardiomyocyte cells. Next, we treated AC16 cells with serum from severe subjects in an acute stage and subsequently analyzed the cell stress and DNA damage markers by western blot. Results indicated that after SARS‐COV‐2 infection, serum with excess immunological factors induces cell stress due to downregulation of p53 and upregulation of HSP70. Moreover, we found that evaluated genetic risk due to increased DNA damage markers, such as γH2Ax and H3K79me2. Altogether, our observations showed that SARS‐COV‐2 could induce stress in cardiomyocytes in an indirect way. SARS‐COV‐2 infection led to macrophage activation, which further secretes pro‐inflammatory cytokines and chemokines; this event eventually results in ongoing activation of vascular endothelial cells, which is a source of reactive oxygen species (ROS) and pro‐inflammatory cytokines.^16^ The ROS, cytokines, and chemokines released to peripheral blood could be cytotoxic to cardiomyocytes, thus inducing cell stress or even more severe injury. HSP70 is an important cell stress response factor,^17^ which is also considered a potential therapeutic target for clinical treatment of SARS‐COV‐2.^18^ Additionally, p53 is a well‐known cell stress response protein, the elevation of p53 usually indicates the occurrence of cell injury under external stress.^19^, ^20^ However, under the treatment of serum from the severe stage, cardiomyocytes cells were not only coping with stress but also undergoing DNA damage. The observations of increased levels of γH2Ax and H3K79me2 suggested an elevated risk of DNA damage. These impacts on cardiomyocytes could be induced by ROS, cytokines, and chemokines released into the blood. The reason that SARS‐COV‐2 was hard to penetrate into AC16 cells was due to the low levels of key receptors. Another limitation of the virus proliferating in cardiomyocytes may be that the viral titers were not high enough in peripheral blood. In this study, we have found a potential elevation of stress in the cardiovascular system in SARS‐COV‐2‐infected subjects. Moreover, coculture of SARS‐COV‐2 and cardiomyocytes suggested that SARS‐COV‐2 can hardly infect cardiomyocytes directly, while the serum from subjects could induce stress and elevated DNA damage risk in cardiomyocytes. Based on these findings, we proposed a theory that SARS‐COV‐2 could induce cardiomyocyte stress or injury in an indirect way.

## 5 CONCLUSION

In summary, our observations indicated that it may be difficult for SARS‐COV‐2 to either infect or proliferate in cardiomyocytes. Additionally, SARS‐COV‐2 still could induce apoptosis of lung epithelial cells via activation of macrophages. The apoptotic epithelial cells release high levels of chemokines and cytokines into the blood, which influences the cardiomyocytes. Although the cardiovascular sequelae of SARS‐COV‐2 infection still need further investigation, our findings revealed an indirect way for SARS‐COV‐2 to damage our cardiovascular system, which suggests a risk of cardiovascular disease even after recovery from SARS‐COV‐2 infection.

## AUTHOR CONTRIBUTIONS

Houqing Zhou collected serum samples and measured serum levels of hs‐cTnT and NT‐proBNP. Xiaohu Ren analyzed the data and wrote the manuscript. Yang Yang performed the “Indirect cell immunofluorescence assay”, Benhong Xu conducted cellular experiments and western blot analysis. Yichong Li revised the manuscript. Shisong Fang and Jianjun Liu designed the research study and provided technical support.

## CONFLICTS OF INTEREST

The authors declare no conflicts of interest.

## Supporting information

Supporting information.

Supporting information.

Supporting information.

Supporting information.

## Data Availability

The raw/processed data required to reproduce these findings cannot be shared at this time as the data also forms part of an ongoing study

## References

[1] Sharma O , Sultan AA , Ding H , Triggle CR . A review of the progress and challenges of developing a vaccine for COVID‐19. Front Immunol. 2020;11:585354. 33163000 10.3389/fimmu.2020.585354 PMC7591699

[2] Harrison AG , Lin T , Wang P . Mechanisms of SARS‐CoV‐2 transmission and pathogenesis. Trends Immunol. 2020;41(12):1100‐1115. 33132005 10.1016/j.it.2020.10.004 PMC7556779

[3] Puelles VG , Lütgehetmann M , Lindenmeyer MT , et al. Multiorgan and renal tropism of SARS‐CoV‐2. N Engl J Med. 2020;383(6):590‐592. 32402155 10.1056/NEJMc2011400 PMC7240771

[4] Hafiane A . SARS‐CoV‐2 and the cardiovascular system. Clin Chim Acta. 2020;510:311‐316. 32681935 10.1016/j.cca.2020.07.019 PMC7363601

[5] Lindner D , Fitzek A , Bräuninger H , et al. Association of cardiac infection with SARS‐CoV‐2 in confirmed COVID‐19 autopsy cases. JAMA Cardiol. 2020;5(11):1281‐1285. 32730555 10.1001/jamacardio.2020.3551 PMC7385672

[6] Puntmann VO , Carerj ML , Wieters I , et al. Outcomes of cardiovascular magnetic resonance imaging in patients recently recovered from coronavirus disease 2019 (COVID‐19). JAMA Cardiol. 2020;5(11):1265‐1273. 32730619 10.1001/jamacardio.2020.3557 PMC7385689

[7] Xiao HH , Wang X , Xu Y , Wang C . [Research advances in cardiovascular system damage caused by SARS‐CoV‐2 in children]. Zhongguo Dang Dai Er Ke Za Zhi. 2020;22(4):299‐304. 32312365 10.7499/j.issn.1008-8830.2003086 PMC7389691

[8] Madjid M , Safavinaeini P , Solomon SD , Vardeny O . Potential effects of coronaviruses on the cardiovascular system: a review. JAMA Cardiol. 2020;5(7):831‐840. 32219363 10.1001/jamacardio.2020.1286

[9] Ragab D , Salah Eldin H , Taeimah M , Khattab R , Salem R . The COVID‐19 cytokine storm; what we know so far. Front Immunol. 2020;11:1446. 32612617 10.3389/fimmu.2020.01446 PMC7308649

[10] Soy M , Keser G , Atagunduz P , Tabak F , Atagunduz I , Kayhan S . Cytokine storm in COVID‐19: pathogenesis and overview of anti‐inflammatory agents used in treatment. Clin Rheumatol. 2020;39(7):2085‐2094. 32474885 10.1007/s10067-020-05190-5 PMC7260446

[11] Jaiswal M , LaRusso NF , Burgart LJ , Gores GJ . Inflammatory cytokines induce DNA damage and inhibit DNA repair in cholangiocarcinoma cells by a nitric oxide‐dependent mechanism. Cancer Res. 2000;60(1):184‐190. 10646872

[12] Bojkova D , Wagner JUG , Shumliakivska M , et al. SARS‐CoV‐2 infects and induces cytotoxic effects in human cardiomyocytes. Cardiovasc Res. 2020;116(14):2207‐2215. 32966582 10.1093/cvr/cvaa267 PMC7543363

[13] Bavishi C , Maddox TM , Messerli FH . Coronavirus Disease 2019 (COVID‐19) infection and renin angiotensin system blockers. JAMA Cardiol. 2020;5(7):745‐747. 32242890 10.1001/jamacardio.2020.1282

[14] Liu L , Liu W , Nie L , et al. Study design and baseline characteristics of Shenzhen ageing‐related disorder cohort in China. BMJ Open. 2020;10(6):e034317. 10.1136/bmjopen-2019-034317 PMC7307537 32565452

[15] Liu C , Mendonça L , Yang Y , et al. The architecture of inactivated SARS‐CoV‐2 with postfusion spikes revealed by cryo‐EM and cryo‐ET. Structure. 2020;28(11):1218‐1224 e1214 33058760 10.1016/j.str.2020.10.001 PMC7557167

[16] Morris G , Bortolasci CC , Puri BK , et al. The pathophysiology of SARS‐CoV‐2: a suggested model and therapeutic approach. Life Sci. 2020;258:118166. 32739471 10.1016/j.lfs.2020.118166 PMC7392886

[17] Radons J . The human HSP70 family of chaperones: where do we stand? Cell Stress Chaperones. 2016;21(3):379‐404. 26865365 10.1007/s12192-016-0676-6 PMC4837186

[18] Rebe C , Ghiringhelli F , Garrido C . Can the hyperthermia‐mediated heat shock factor/heat shock protein 70 pathway dampen the cytokine storm during SARS‐CoV‐2 infection? *Br J Pharmacol*. 2020 10.1111/bph.15343 33314076

[19] Haronikova L , Olivares‐Illana V , Wang L , Karakostis K , Chen S , Fahraeus R . The p53 mRNA: an integral part of the cellular stress response. Nucleic Acids Res. 2019;47(7):3257‐3271. 30828720 10.1093/nar/gkz124 PMC6468297

[20] Gould KA , Nixon C , Tilby MJ . p53 elevation in relation to levels and cytotoxicity of mono‐ and bifunctional melphalan‐DNA adducts. Mol Pharmacol. 2004;66(5):1301‐1309. 15308759 10.1124/mol.104.000596

