# Supplementary figures and images for: An alternative way of SARS‐COV‐2 to induce cell stress and elevated DNA damage risk in cardiomyocytes without direct infection
Source: Immun Inflamm Dis. 2022 Jun 10;10(7):e638. doi: 10.1002/iid3.638 (PMC9186334; doi:10.1002/iid3.638)

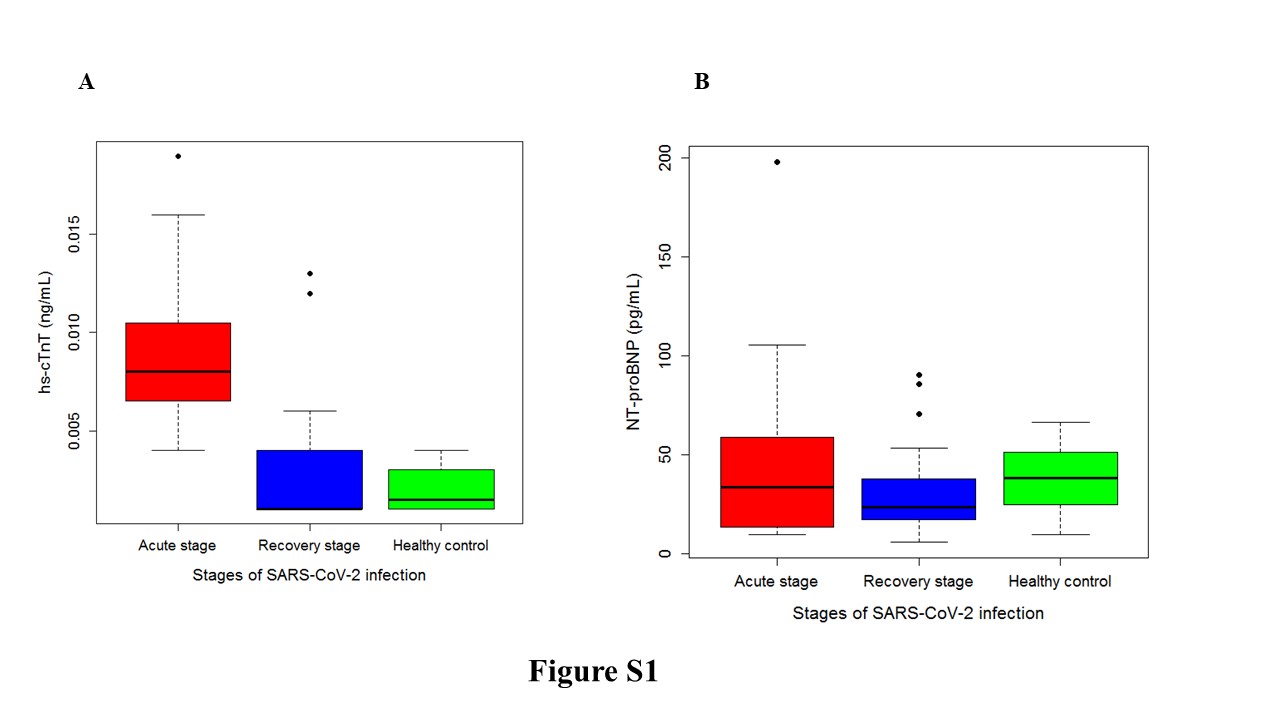

Supplement: Supplementary file 1 — Supporting information. [file IID3-10-0-s002.JPG]

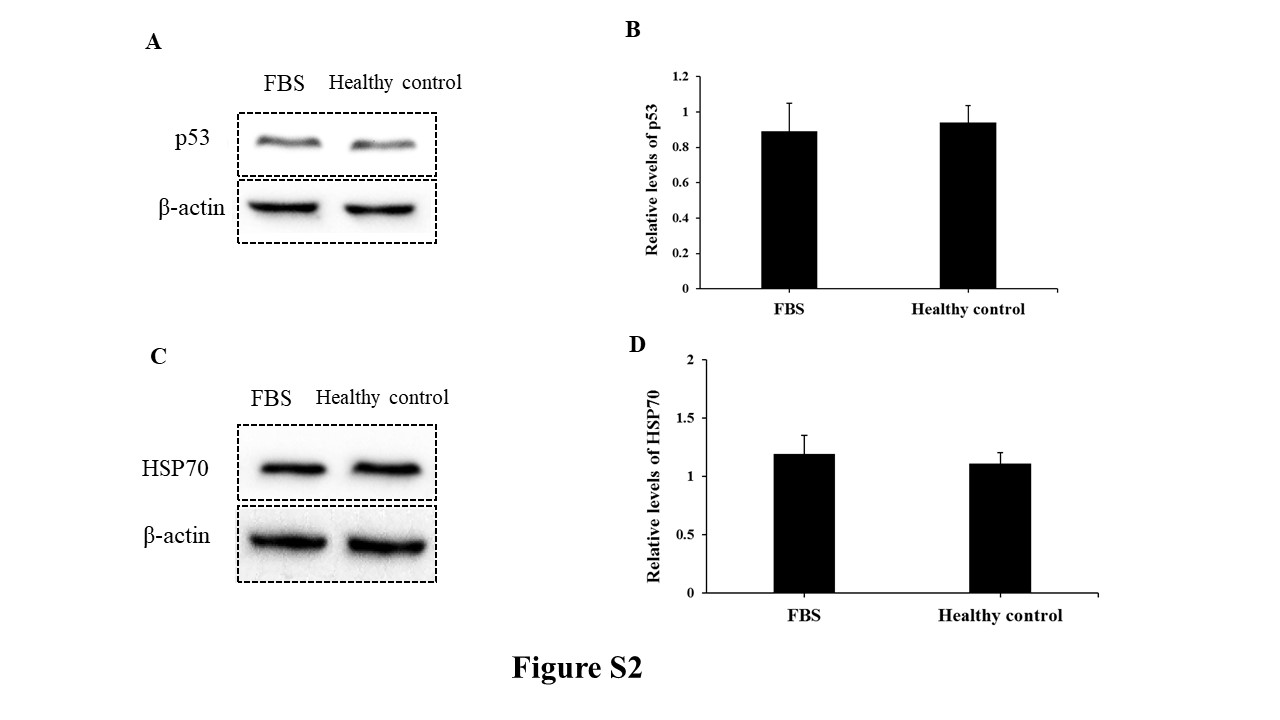

Supplement: Supplementary file 2 — Supporting information. [file IID3-10-0-s004.JPG]

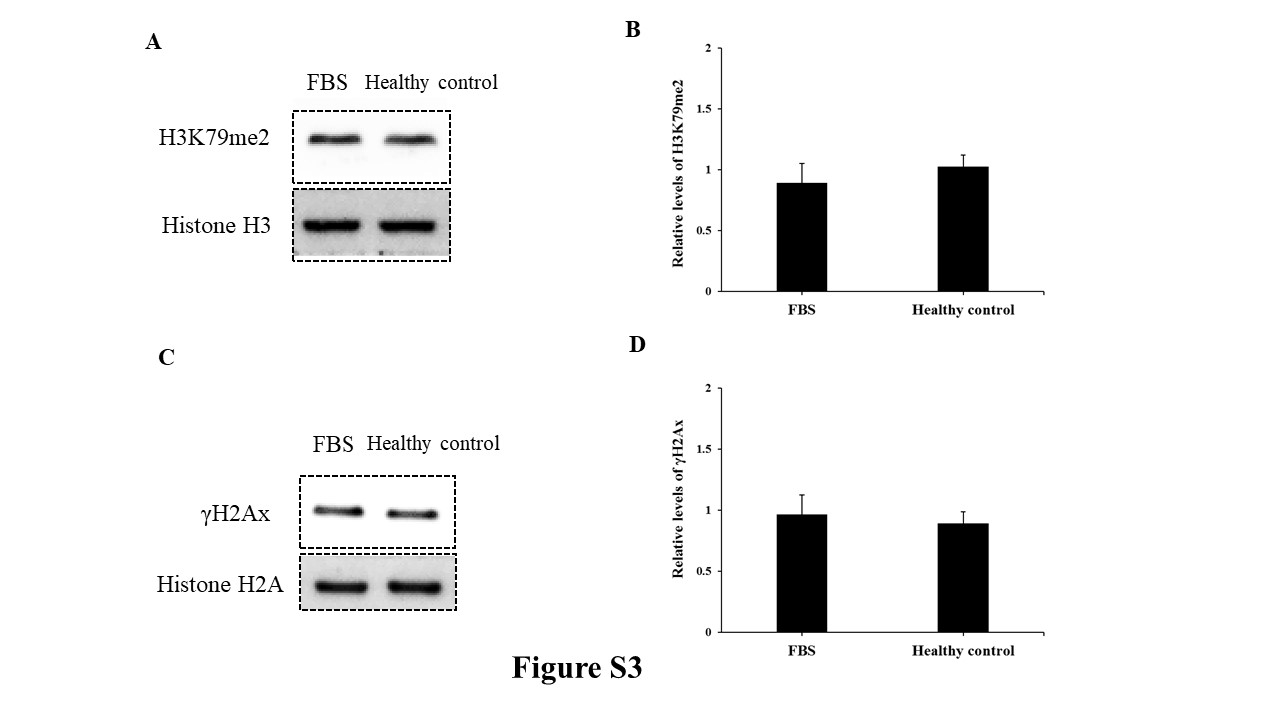

Supplement: Supplementary file 3 — Supporting information. [file IID3-10-0-s001.JPG]
