# Supplementary material for: An alternative way of SARS‐COV‐2 to induce cell stress and elevated DNA damage risk in cardiomyocytes without direct infection
Source: Immun Inflamm Dis. 2022 Jun 10;10(7):e638. doi: 10.1002/iid3.638 (PMC9186334; doi:10.1002/iid3.638)
Supplement: Supplementary file 4 — Supporting information. [file IID3-10-0-s003.docx]

**Fig. S1 Evaluation of hs-cTnT and NT-proBNP in serum of healthy controls**

The serum levels of hs-cTnT and NT-proBNP were measured by ELISA. A:box-plot of hs-cTnT in acute stage, recovery stage and healthy control, black line represents median, there were no significant differences of hs-cTnT between healthy controls and recovery stage (healthy controls n=19,acute stage n=18, recovery stage n=18); B:box-plot of NT-proBNP in acute stage, recovery stage and healthy control, black line represents median, there were no significant differences of hs-cTnT between healthy controls and recovery stage (healthy controls n=19,acute stage n=18, recovery stage n=18).

**Fig. S2 No changes of p53 and HSP70 in AC16 cells between the treatments of FBS and serum from healthy controls**

The relative levels of p53 and HSP70 in AC16 cells were evaluated by Western-blot analysis after treatment of FBS and serum from healthy controls for 24 hours. A: protein band of p53; B: the relative level of p53, bar graphics reflect the mean ± SD at least three independent experiments; C: protein band of HSP70; D: the relative level of HSP70, bar graphics reflect the mean ± SD at least three independent experiments.

**Fig. S3 No changes of γH2Ax and H3K79me2 in AC16 cells between the treatments of FBS and serum from healthy controls**

The relative level of γH2Ax and H3K79me2 in AC16 cells were evaluated by Western-blot analysis after treatment of FBS and serum from healthy controls for 24 hours. A: protein band of γH2Ax; B: the relative level of γH2Ax, bar graphics reflect the mean ± SD at least three independent experiments; C: protein band of H3K79me2; D: the relative level of H3K79me2, bar graphics reflect the mean ± SD at least three independent experiments.

**Table 1S Statistical analysis of clinical parameters**

| Clinical parameters | Asymptomatic | Moderate | Severe | *P* |
| --- | --- | --- | --- | --- |
| n | 4 | 9 | 5 |  |
| male (%) | 1 ( 25.0) | 4 ( 44.4) | 2 ( 40.0) | 0.801 |
| Female(%) | 3 ( 75.0) | 5 ( 55.6) | 3 ( 60.0) |  |
| Age (mean (SD)) | 19.25 (19.36) | 36.11 (14.16) | 61.60 (12.24) | 0.002 |
| Systolic (mean (SD)) | 119.75 (17.48) | 123.78 (8.94) | 146.40 (24.21) | 0.04 |
| Diastolic (mean (SD)) | 76.75 (23.26) | 82.11 (6.97) | 87.20 (13.37) | 0.526 |
| Heart rate (mean (SD)) | 86.00 (11.89) | 87.67 (9.58) | 92.60 (6.58) | 0.538 |
